# Are growing pains a parasomnia?

**DOI:** 10.1186/1546-0096-9-S1-P225

**Published:** 2011-09-14

**Authors:** F Aeschlimann, H Werner, O Jenni, RK Saurenmann

**Affiliations:** 1 Rheumatology, University of Zürich, Switzerland; 2 Developmental Medicine Children’s Hospital, University of Zürich, Switzerland

## Background

The so called growing pains (GP) are affecting 4-37% of all children with a peak incidence in the preschool ages. Although parasomnias (e.g.sleep terrors, sleep talking, sleep walking) share several features with GP such as the age at onset, the daytime of appearance, the self-limited course and the absence of symptoms on the following day, the 2 conditions have not been linked so far.

## Objective

To analyse the pain characteristics of children with GP and compare sleep characteristics of children with and without GP in order to investigate the possibility that GP constitute a parasomnia.

## Patients and Methods

The parents of 58 children with a diagnosis of GP filled a questionnaire about the characteristics of the GP and the sleep of their children. The study group was subdivided in 2 groups according to the time of pain onset: “evening GP” occurring already before bedtime, and “night GP”occurring only after falling asleep. 38 children from a study about children’s sleep pattern served as a control cohort.

## Results

Children with GP had more difficulties waking up in the morning (p<0.0001), had more difficulties with re-sleeping after waking up (p<0.0001), had a lower overall sleep quality (p=0.0002), more commonly used a cuddly toy (p=0.002) and more often had sleep terrors (p=0.005). In a multivariate analysis the factors wake-up difficulties, re-sleeping difficulties, sleep terrors and transitional object remained independently significantly associated with GP. 14 children (24%) qualified for the definition of night GP and 16 (28%) had evening GP. Night GP were significantly more common in boys (p=0.009), had fewer pain attacks during one night (p=0.04), were less likely to have their pain attacks following hectic days (p=0.04), had a better overall sleep quality (p=0.049) and more commonly also had sleep terrors (p=0.1) than children with evening GP. In the multivariate analysis the factors gender, sleep terrors and occurrence after hectic days remained independently significant.

## Conclusion

Children with the so called GP have a disturbed sleep pattern. The highly significant association of growing pains, especially of the night GP variant, with sleep terrors supports the hypothesis of a link between these conditions and warrants further investigations.

